# “Air-Lock” gating mechanism of CsoS1D for metabolite translocation through the α-carboxysome shell

**DOI:** 10.1093/plphys/kiag331

**Published:** 2026-06-03

**Authors:** Quan Wen, Yue Wang, Guo-Can Huang, Hong-Yu Pan, Yue-Yang Tang, Li-Hua Bie, Lu-Ning Liu, Jun Gao

**Affiliations:** Hubei Key Laboratory of Agricultural Bioinformatics, College of Informatics, Huazhong Agricultural University, Wuhan 430070, China; Hubei Key Laboratory of Agricultural Bioinformatics, College of Informatics, Huazhong Agricultural University, Wuhan 430070, China; Hubei Key Laboratory of Agricultural Bioinformatics, College of Informatics, Huazhong Agricultural University, Wuhan 430070, China; Hubei Key Laboratory of Agricultural Bioinformatics, College of Informatics, Huazhong Agricultural University, Wuhan 430070, China; Institute of Systems, Molecular and Integrative Biology, University of Liverpool, Liverpool L69 7ZB, United Kingdom; Hubei Key Laboratory of Agricultural Bioinformatics, College of Informatics, Huazhong Agricultural University, Wuhan 430070, China; Hubei Key Laboratory of Agricultural Bioinformatics, College of Informatics, Huazhong Agricultural University, Wuhan 430070, China; Institute of Systems, Molecular and Integrative Biology, University of Liverpool, Liverpool L69 7ZB, United Kingdom; Hubei Key Laboratory of Agricultural Bioinformatics, College of Informatics, Huazhong Agricultural University, Wuhan 430070, China

## Abstract

Carboxysomes are specialized bacterial microcompartments (BMCs) for CO_2_ assimilation in cyanobacteria and many chemoautotrophs. Selective transport of gas molecules and metabolites across the carboxysome shell plays an essential role in creating a high-CO_2_ environment around Rubisco and ensuring efficient metabolite flux. However, the molecular mechanisms underlying this specific permeability remain elusive. Using integrated computational approaches, including all-atom molecular dynamics (MD) simulations, self-random acceleration MD simulations, umbrella sampling and targeted MD simulations, we systematically investigated the permeation pathways of large anionic metabolites ribulose-1,5-bisphosphate (RuBP) and 3-phosphoglycerate (3-PGA) through the α-carboxysome shell protein CsoS1D, which exhibits a trimer-of-dimer architecture and an enlarged central pore compared with hexameric and pentameric shell proteins. The results indicate that the central pore of CsoS1D serves as the primary conduit for the translocation of bulky metabolites RuBP and 3-PGA and reveal a 3-stage “air-lock” transport mechanism driven by electrostatic interactions. Moreover, the shallow free-energy landscape for channel gating enables the pore to undergo frequent, thermally driven transitions between open and closed states, implementing a conformational selection transport model independent of ligand binding. Our analysis further revealed 5 conserved residues that establish an electrostatic transport pathway within trimeric shell proteins, suggesting that this permeability mechanism represents a generalizable design principle across diverse BMCs. By elucidating shell protein permeability mechanisms at atomic resolution, this study lays the framework for understanding carboxysome physiology and guides the rational engineering of carboxysome permeability to facilitate system-level metabolic modeling and optimization of synthetic carboxysomes for biotechnological applications.

## Introduction

Carboxysomes are a family of bacterial microcompartments (BMCs) in cyanobacteria and many chemoautotrophs, which encapsulate ribulose-1,5-bisphosphate (RuBP) carboxylase/oxygenase (Rubisco) and carbonic anhydrase within a protein shell (Liu 2022; Li et al. 2024). By locally concentrating CO_2_ around Rubisco, carboxysomes form the core of the CO_2_-concentrating mechanism, thereby improving photosynthetic carbon assimilation and microbial fitness under fluctuating environmental conditions (Price et al. 2008; Rae et al. 2013; Kerfeld et al. 2018). To maintain the special interior microenvironment, the proteinaceous shell must balance opposing demands: restricting CO_2_ leakage and undesired metabolites while allowing rapid and sufficient flux of bicarbonate, RuBP and 3-phosphoglycerate (3-PGA) (Yeates et al. 2008; Cai et al. 2009; Huang et al. 2022; Sarkar et al. 2024).

**Figure 1:**
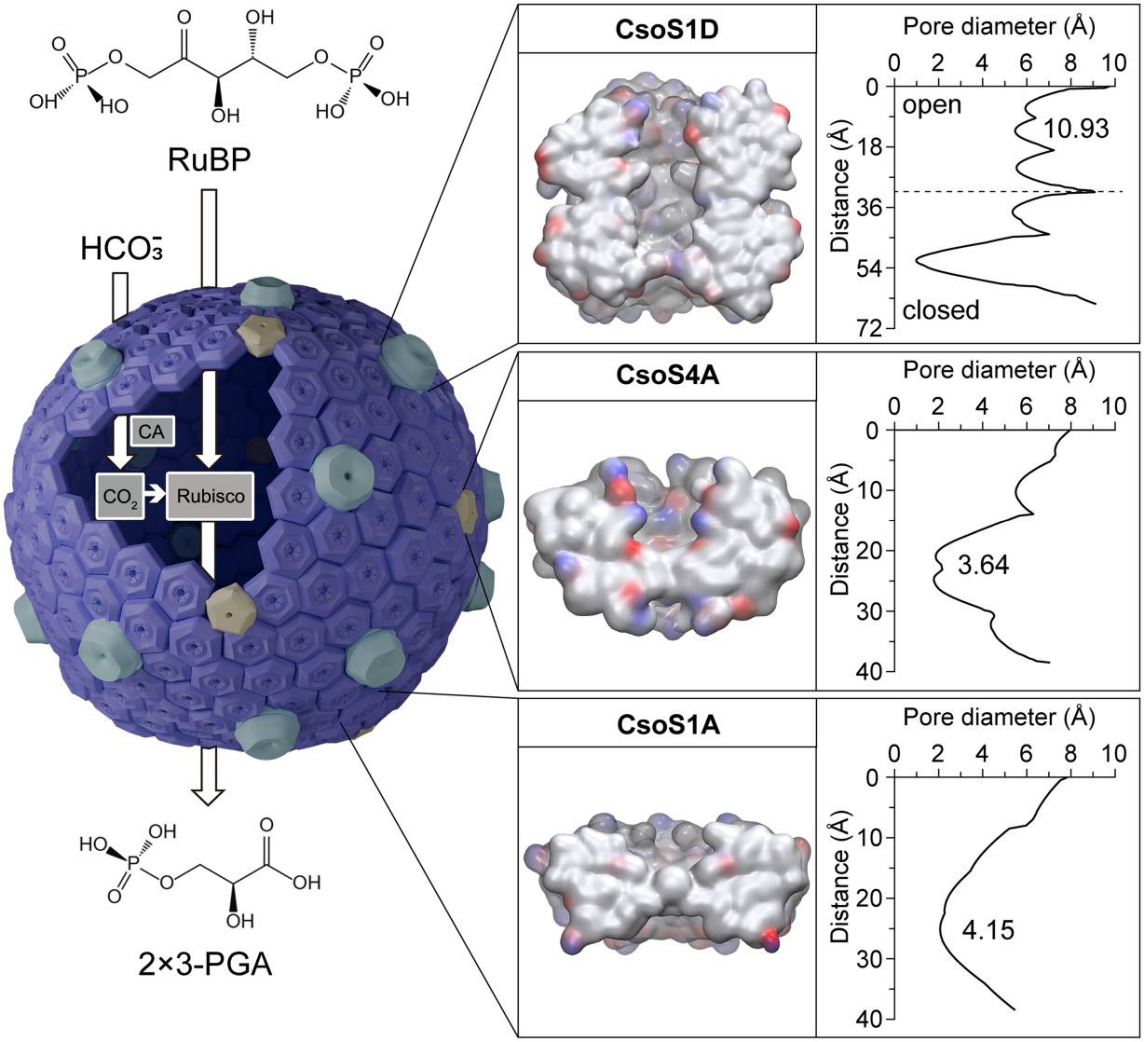
Schematic representation of the α-carboxysome and pore sizes of shell proteins. Left: Model of the α-carboxysome illustrating RuBP influx and 3-PGA efflux. CA, carbonic anhydrase. Right: Structural models of the pore units of CsoS1D (BMC-T, PDB ID: 3FCH), CsoS4A (BMC-P, PDB ID: 2RCF), and CsoS1A (BMC-H, PDB ID: 2EWH), colored to highlight pore geometry and cross-sectional area. The CsoS1D dimer displays 1 open and 1 closed protomer, whereas BMC-P and BMC-H exhibit uniformly narrow pores. The pore diameter in the dimer-of-trimers assembly is plotted as a function of vertical position along the pore axis (right).

The α-carboxysome shell is constructed from several classes of BMC proteins, including hexameric BMC-H (eg CsoS1A), pentameric BMC-P (eg CsoS4A), and trimeric BMC-T (CsoS1D) tandem BMC components (Fig. 1; Tsai et al. 2007; Tanaka et al. 2008; Kerfeld and Melnicki 2016). While BMC-H and BMC-P proteins primarily form flat facets and vertices of the icosahedral shell, the BMC-T family features a 2-domain architecture producing a pseudo-hexameric unit (Sutter et al. 2017; Ni et al. 2023; Wang et al. 2024; Correa et al. 2025) and exhibits a low copy in BMCs (Yang et al. 2020; Sun et al. 2022, 2025).

Previous studies have indicated that the central pores of BMC shell proteins serve as selective conduits for key metabolites including bicarbonate, RuBP, and 3-PGA; this selective permeability is achieved through a synergistic mechanism of size exclusion, which limits passage based on steric hindrance, and electrostatic filtering, which utilizes charged residues within the pore lumen to attract or repel specific metabolites (Kinney et al. 2011; Mahinthichaichan et al. 2018; Faulkner et al. 2020; Sarkar et al. 2024). The crystal structures of CsoS1D revealed an enlarged pore size and asymmetric assemblies, in which 1 protomer adopts an open conformation and the other a closed conformation, suggesting dynamic gating at the level of individual pores (Klein et al. 2009; Roberts et al. 2012). Despite these structural insights, the mechanistic basis of bulky RuBP and 3-PGA metabolite transport through α-carboxysome shell proteins remains unclear. In particular, 3 key questions remain: (i) Is CsoS1D the principal transport route for RuBP and 3-PGA compared with other shell proteins in the α-carboxysome? (ii) What is the free-energy landscape for RuBP and 3-PGA permeation, and how does it constrain flux directionality and mitigates product inhibition? (iii) How are conformational switching and local electrostatics integrated to form controllable molecular gates for RuBP and 3-PGA?

In this study, we implemented an integrative computational strategy to investigate the permeability mechanisms of CsoS1D by combining all-atom molecular dynamics (MD) simulations, self-random acceleration MD (s-RaMD-MD) simulations, and targeted MD (TMD) simulations coupled with umbrella sampling. Our results support a mechanistic model in which CsoS1D operates as an “air-lock” valve, coordinating electrostatic capture and release of RuBP and 3-PGA with alternating openings of the outer and inner pore faces. This study provides an atomic-level view of carboxysome shell permeability and defines specific residues and energetic features that can be targeted in future mutagenesis and synthetic biology to engineer shell proteins for modified metabolite flux within synthetic carboxysomes. More broadly, CsoS1D belong to a widely distributed family of tandem BMC-domain shell proteins found across diverse BMCs, including β-carboxysomes and numerous metabolosomes (Melnicki et al. 2021). This evolutionary perspective underscores the relevance of our findings beyond α-carboxysomes and highlights BMC-T proteins as tunable, conserved permeability modules for engineering synthetic BMCs.

## Results

### CsoS1D serves as the primary conduit for RuBP and 3-PGA translocation

To compare the intrinsic permeability potential of different shell proteins to accommodate RuBP and 3-PGA, we examined the pores of CsoS1D, CsoS4A, and CsoS1A using the structural models derived from the crystal structures of individual shell proteins (Figs. 1, S1 and S2). The pore diameters were quantified using the MolAICal software (Bai et al. 2021). Structural analysis revealed that CsoS1A (PDB ID: 2EWH) and CsoS4A (PDB ID: 2RCF) proteins possess central apertures that are highly constrained by steric hindrance for larger molecules (Tsai et al. 2007; Tanaka et al. 2008), with pore widths comparable to those of BMC-H and BMC-P proteins across carboxysomes and metabolosomes. CsoS1A displays minimal diameters ranging from 3.8 to 4.6 Å, while CsoS4A exhibits minimal diameters between 3.6 and 5.8 Å. Geometric analysis based on hydrated diameters (3-PGA, ∼3.8 Å; RuBP, ∼4.8 Å) is consistent with a remarkable steric barrier of these bulky metabolites from hexameric and pentameric shell protein pores, suggesting that CsoS1D may offer a more accessible route.

The available CsoS1D trimer-of-dimer structures to date exhibit either a “fully closed” (PDB ID: 7DHQ) (Tan et al. 2021) or an asymmetric “one-open/one-closed” (PDB ID: 3FCH, 3F56) configuration (Klein et al. 2009; Fig. S3). We chose the higher-resolution PDB ID: 3FCH structure (2.20 Å, one-open/one-closed) from *Prochlorococcus marinus* MED4 as the model in this study, which allows for a more realistic representation of the physiological asymmetry inherent to dynamic transport process. The open trimer of CsoS1D displays a substantially larger cross-section and more solvent-accessible pore (10.9 to 12.4 Å), whereas the closed trimer forms a compact, occluded channel (Klein et al. 2009; Fig. 1).

To test whether static structural differences translate into functional permeation behaviors, s-RaMD-MD simulations (Mao et al. 2024) were performed on 6 protein-ligand combinations: 3-PGA and RuBP each interacting with CsoS1D, CsoS4A, and CsoS1A. Ligands were initially placed 15 Å from the pore entrance, and subjected to 2 random acceleration strengths (0.2 and 0.3 kcal mol^−1^ Å^−1^ amu^−1^) to overcome kinetic barriers. For each protein-ligand combination, 50 independent 1-ns trajectories were generated, yielding a total of 300 s-RaMD-MD simulations. Successful translocation events within the 1-ns sampling window were counted to estimate passage probabilities (Table 1).

**Table 1:** Passage probability (%) of 3-PGA and RuBP through the pores of CsoS1D, CsoS1A, and CsoS4A determined by s-RaMD-MD simulations.

Acceleration system	*a* = 0.2^a^		*a* = 0.3^a^	
	Passage events	Probability (%)	Passage events	Probability (%)
3-PGA-CsoS1A	0/50	0	15/50	30
RuBP-CsoS1A	0/50	0	1/50	2
3-PGA-CsoS4A	0/50	0	6/50	12
RuBP-CsoS4A	0/50	0	2/50	4
3-PGA-CsoS1D	28/50	56	35/50	70
RuBP-CsoS1D	23/50	46	26/50	52

Contains ligand-protein molecular systems, acceleration (0.2 or 0.3 kcal mol^−1^ Å^−1^ amu^−1^), number of successful permeation events/number of trials and passage probability (%).

^a^Accelerations are in kcal mol^−1^ Å^−1^ amu^−1^.

Molecule translocation efficiency revealed a clear functional hierarchy among the shell proteins. At low acceleration (0.2 kcal mol^−1^ Å^−1^ amu^−1^), CsoS1A and CsoS4A were proved to be essentially non-permeable to both 3-PGA and RuBP. In contrast, the open protomer pore architecture of CsoS1D enabled efficient metabolite passage (56% of trajectories for 3-PGA and 46% for RuBP) (Table 1; Movies S1 and S2). This selectivity pattern was strengthened at higher acceleration forces: CsoS1D exhibited remarkably higher permeation probabilities (3-PGA 70%; RuBP 52%), whereas CsoS1A and CsoS4A remained predominantly impermeable or weakly permissive (CsoS1A: 3-PGA 30%, RuBP 2%; CsoS4A: 3-PGA 12%, RuBP 4%). Combined with structural pore diameter analysis (Fig. 1), these kinetic data suggest that CsoS1D serves as a potential transport conduit for bulky 3-PGA and RuBP across the α-carboxysome shell, despite its low abundance. Notably, the relatively smaller anionic metabolite 3-PGA shows greater permeability than RuBP through the central pore of CsoS1D, suggesting inherent permeability selectivity which may avoid RuBP leakage while allowing efficient 3-PGA export.

### Three-stage free-energy landscape for metabolite transport

**Figure 2:**
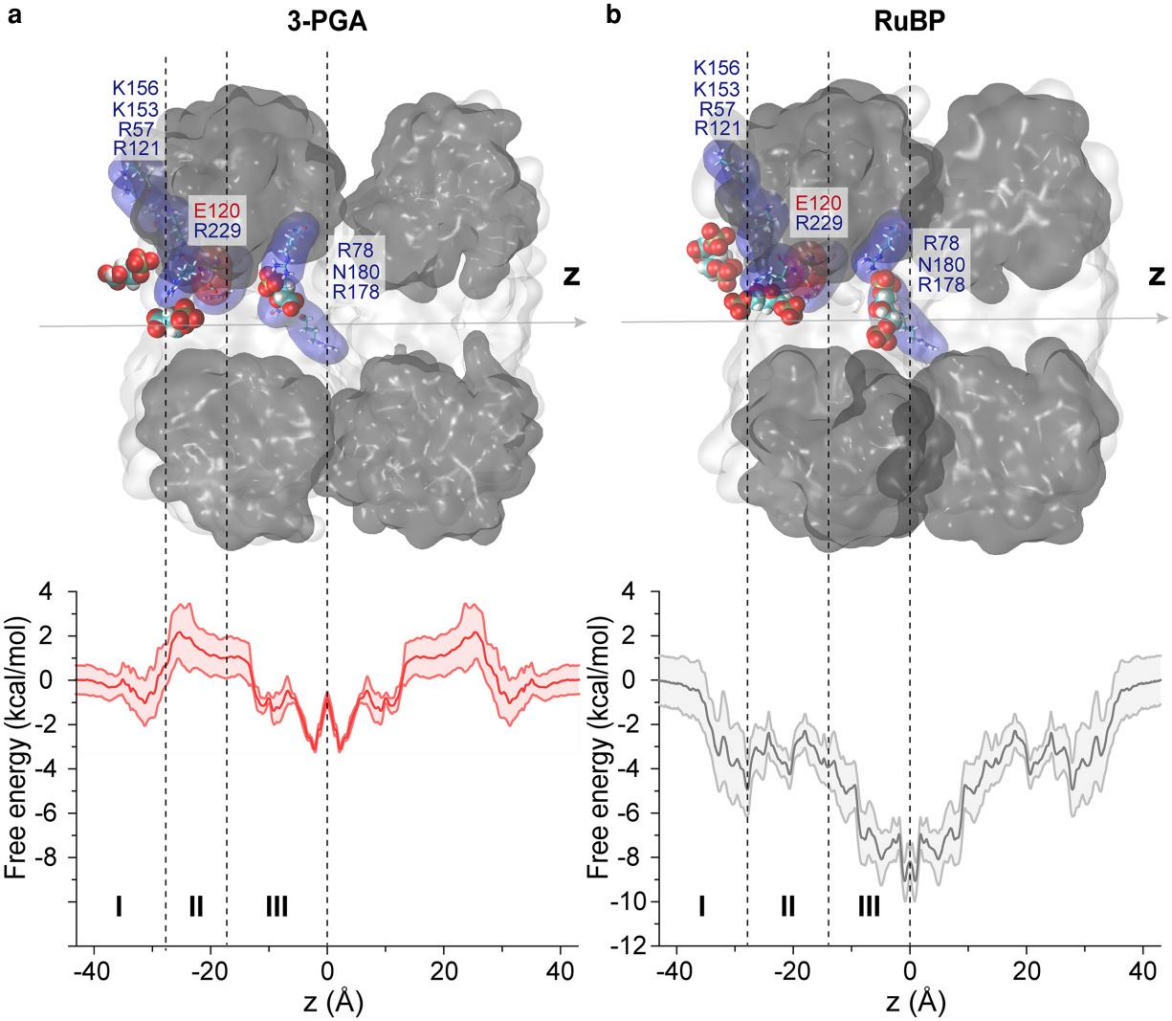
Free-energy landscapes of 3-PGA and RuBP permeation through CsoS1D. a) Visualization of the CsoS1D pore and the corresponding free-energy profile of 3-PGA projected along the pore axis. b) Visualization of the CsoS1D pore and the corresponding free-energy profile of RuBP. Negatively and positively charged residues are shown in red and blue, respectively. Vertical dashed lines divide the permeation pathway into 3 stages: Stage I, initial metabolite entry accompanied by a decrease in free energy; Stage II, traversal of the primary energy barrier formed by E120; and Stage III, further free-energy reduction and stabilization near the CsoS1D dimer interface.

Then, we employed umbrella sampling simulations to reconstruct the free-energy landscapes of RuBP and 3-PGA permeation through CsoS1D, using initial configurations derived from s-RaMD-MD trajectories. The ligand center-of-mass (COM) projection along the pore axis served as the reaction coordinate, yielding well-converged free-energy profiles (Fig. S4). Given the asymmetric nature of the simulated CsoS1D trimer-of-dimer structure, the free-energy profile obtained from the open trimer was mirrored to construct a continuous, symmetrical energy landscape representing a complete *trans*-shell passage (Fig. 2).

The resulting free-energy profiles reveal substantially disparate barriers for the 2 metabolites: 3-PGA exhibits a markedly lower free-energy minimum (∼ −5 kcal mol^−1^) than RuBP (∼ −9 kcal mol^−1^), indicating a more favorable thermodynamic translocation of 3-PGA through CsoS1D (Fig. S4). This energetic hierarchy implies efficient spontaneous export of 3-PGA from the carboxysome lumen, whereas RuBP permeation requires additional thermodynamic or kinetic driving forces to overcome the higher barrier. Notably, this trend is consistent with the s-RaMD-MD permeation statistics (Table 1), indicating the concordance between thermodynamic and accelerated kinetic sampling and confirming that 3-PGA possesses an intrinsic transport advantage through CsoS1D compared with RuBP.

**Figure 3:**
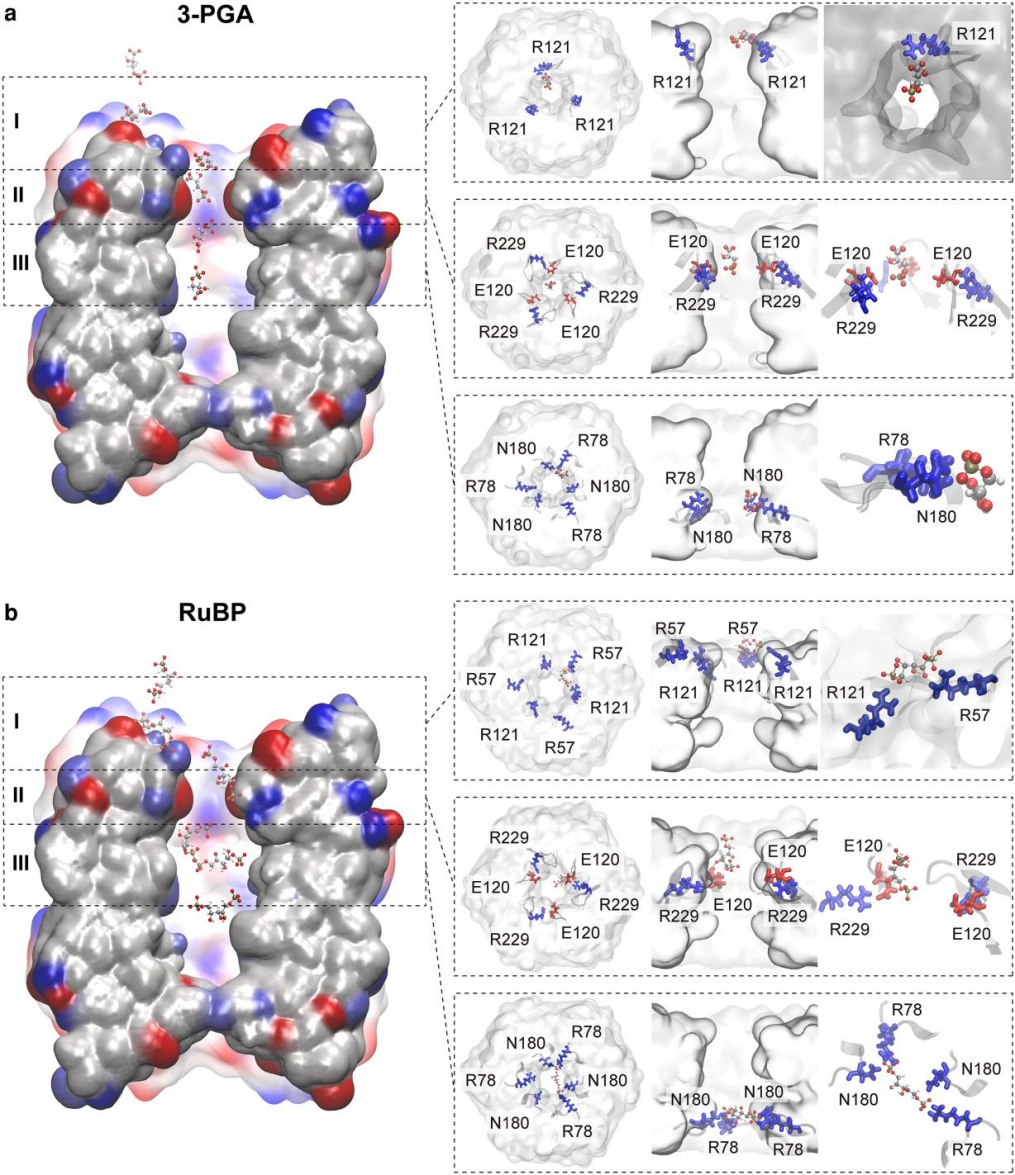
Three-stage structural mechanism of metabolite translocation through CsoS1D. a) Representative snapshots and schematic diagrams illustrating 3-PGA permeation through the CsoS1D pore, highlighting the 3 stages identified in Fig. 2: Stage I, metabolite capture and guidance at the pore entrance; Stage II, passage through the E120 gate; Stage III, transient stabilization within the pore followed by release to the opposite side. b) Representative snapshots and schematic diagrams of RuBP permeation through the CsoS1D pore, showing the same 3-stage translocation process, with distinct metabolite-residue interactions at the entrance and shared gating and stabilization mechanisms in the subsequent stages.

Despite their different barrier heights, both 3-PGA and RuBP exhibit a conserved 3-stage free-energy profile during permeation across CsoS1D (Fig. 2). This process involves: (i) metabolite capture at the pore entrance, (ii) passage through the E120 electrostatic ring, and (iii) release from the interior electropositive cluster at the exit. Analysis of the molecular configurations along the reaction coordinate reveals that transport through the 3 stages is predominantly governed by electrostatic interactions (Fig. 3). This mechanistic architecture, wherein anionic metabolites are sequentially captured, gated, and released via electrostatic interactions, defines a unified 3-stage transport framework. This architecture is consistent with previous electrostatic focusing models proposed for carboxysome shell pores, in which charged pore linings and potential gradients were shown to drive the selective transit of small anionic metabolites through hexameric shell proteins, such as CsoS1A and CcmK2 (Mahinthichaichan et al. 2018; Faulkner et al. 2020). Collectively, these data reveal that CsoS1D mediates the transport of 3-PGA and RuBP through a unified, electrostatic-driven 3-stage mechanism governed by electrostatic interactions.

#### Stage I: entrance binding and electrostatic interactions mediate metabolite capture

The first stage corresponds to metabolite entry from the bulk solution into the pore entrance and is dominated by electrostatic interactions with positively charged residues (R57, R121, K153, and K156) (Figs 2 and 3). In this region, the free energy decreased by ∼ −1 kcal mol^−1^ for 3-PGA and by ∼ −5 kcal mol^−1^ for RuBP, indicating a stronger initial stabilization of RuBP. This difference arises from the distinct binding modes at the entrance, where R57, R121, K153, and K156 interact with the metabolite phosphate groups via electrostatic attraction and hydrogen bonding. Owing to its single phosphate group and compact geometry, 3-PGA typically interacts with only 1 or 2 positively charged residues, most often with a single R121 side chain, resulting in weak stabilization (Fig. 3a). In contrast, RuBP, with 2 terminal phosphate groups, can simultaneously engage multiple basic residues, including R121 and R57 from different subunits, forming an extensive interaction network and a deeper free-energy minimum (Fig. 3b). This differential stabilization has critical mechanistic consequences: while stronger entrance binding of RuBP initially favors capture, it simultaneously increases the thermodynamic cost of subsequent escape from this state, creating a kinetic bottleneck for downstream translocation.

#### Stage II: Passage through the E120 electrostatic gate

The second stage involves metabolite transit through the narrow constriction formed by a ring of E120 residues, which constitutes the dominant electrostatic barrier along the permeation pathway. Both 3-PGA and RuBP encounter a similar free-energy barrier of ∼3 kcal mol^−1^, arising from electrostatic repulsion between their phosphate groups and the E120 carboxylates (Figs. 2 and 3). Importantly, the effective height of this barrier is modulated by R229, which is positioned adjacent to the E120 ring and engages in electrostatic attraction with E120, partially compensating for the local negative electrostatic potential generated by E120 and thereby fine-tuning the barrier experienced by anionic substrates. RuBP approaches the E120 barrier from a lower energy state owing to its stronger entrance stabilization and exhibits a characteristic double-barrier feature, reflecting the sequential passage of its 2 phosphate groups through the pore. Notably, similar electrostatically induced free-energy elevations have been reported for other phosphorylated metabolites, such as glyceraldehyde-3-phosphate (G3P) and dihydroxyacetone phosphate (DHAP), during permeation through BMC-T shell proteins, where negatively charged residues lining the pore create transient energetic barriers to anionic substrates (Raza et al. 2025). These results suggest that E120-mediated electrostatic gating represents a conserved selectivity mechanism for phosphorylated metabolites.

#### Stage III: Internal electrostatic stabilization

The third stage involves ligand migration toward the trimer-of-dimer interface, where a cluster of positively charged and polar residues (R78, R178, and N180) establishes an internal electropositive binding pocket. After successful passage through the E120 barrier, metabolites encounter stabilizing electrostatic interactions with this internal cluster, generating a secondary free-energy well. The depth of this stabilization is metabolite-dependent: RuBP, which carries 2 terminal phosphate groups and extended geometry, is commonly observed in the lowest-energy configurations by simultaneously engaging R78-N180 residue pairs from 2 adjacent protein chains, thereby forming multivalent electrostatic contacts and a deep free-energy minimum (∼ −9 kcal mol^−1^). In contrast, 3-PGA interacts with only a single R78-N180 pair from 1 chain, producing a substantially shallower minimum (∼ −3 kcal mol^−1^). Thus, this stage corresponds to a transient, electrostatically anchored internal binding state whose thermodynamic stability is directly proportional to metabolite charge and geometric compatibility with the binding pocket architecture.

### Small free-energy barrier mediates the closed-to-open conformational change

**Figure 4:**
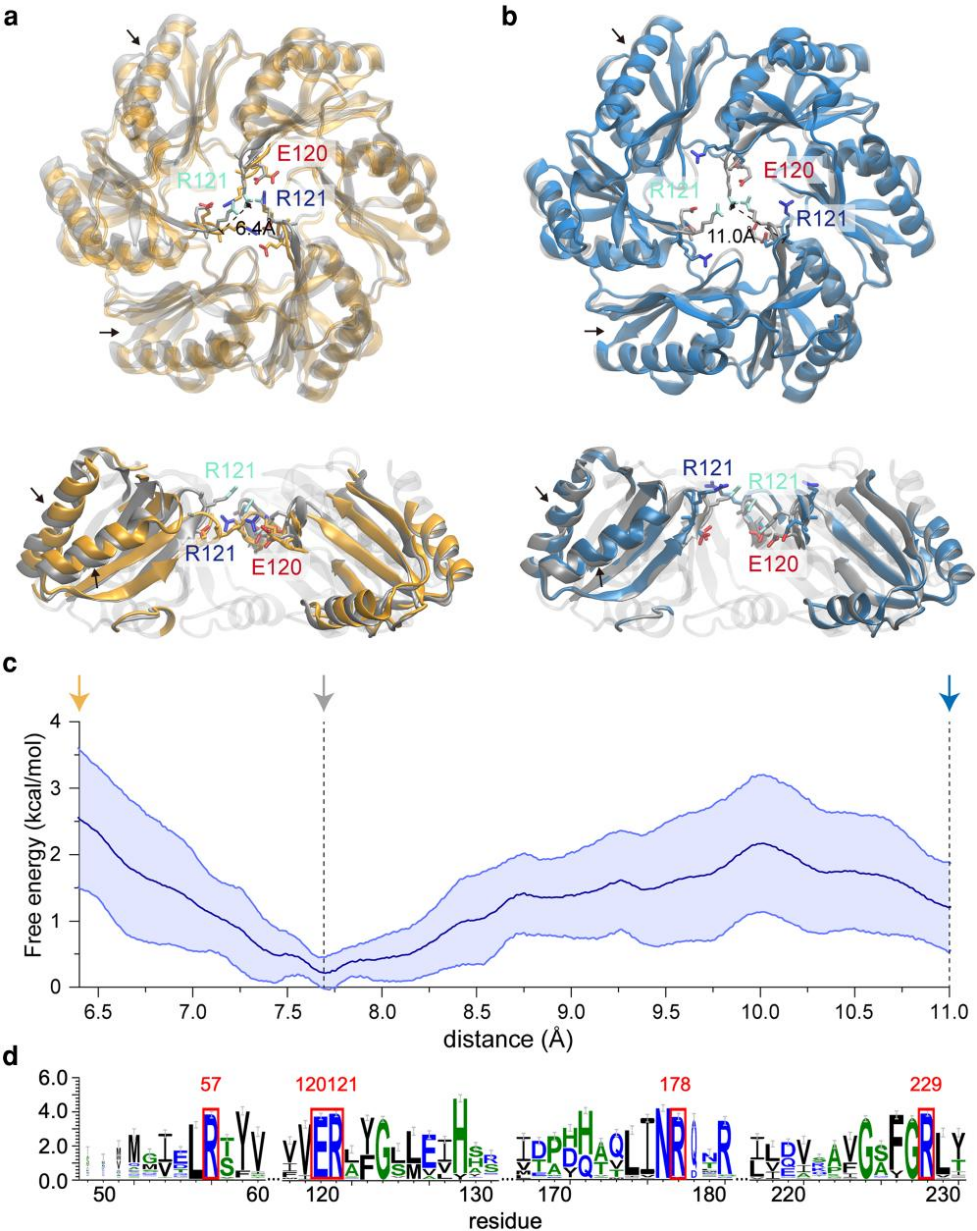
Conformational gating mechanism mediated by the E120-R121 molecular latch. a) Superposition of the initial closed state (orange) and the semi-open intermediate (gray), illustrating global backbone rearrangements while the side chain of R121 remains in a closed conformation. b) Superposition of the targeted open state (blue) and the semi-open intermediate (gray), showing that the backbone of the semi-open state closely resembles the open conformation, whereas the side chain of R121 and adjacent loop regions remain closed. Key residues in (a) and (b) are shown as sticks: E120 (red) and R121 (blue). The reaction coordinate is defined as the distance between the COM of the backbone C and N atoms of residue R121 in a single subunit and the corresponding COM in the trimeric reference structure. c) Free-energy profile of the conformational transition reconstructed from TMD and umbrella sampling. The reaction coordinate is defined by the displacement of the R121 backbone. An energy minimum at ∼7.7 Å (gray arrow) corresponds to the semi-open intermediate, which is separated from the closed (∼6.4 Å, orange arrow) and open (∼11.0 Å, blue arrow) states by relatively low free-energy barriers. These features indicate a dynamically gated channel in which disruption of the E120–R121 interaction is readily accessible under thermal fluctuations. Subunit-resolved profiles are shown in Fig. S5. d) Sequence alignment of CsoS1D homologs presented by WebLogo, showing that the 5 key residues (R57, E120, R121, R178, and R229) critical for the “air-lock” mechanism are highly conserved among diverse taxa, and thereby, supporting the presence of a multi-stage electrostatic pathway for metabolite passage. Full sequence alignment results are shown in Fig. S6.

To probe the molecular mechanism governing channel gating, TMD simulations were performed to generate a continuous closed-to-open transition, using closed- and open-side conformations resolved in the crystal structure of CsoS1D (PDB ID: 3FCH) (Klein et al. 2009; Movies S3 and S4). Umbrella sampling was conducted along a distance-based reaction coordinate, defined as the separation between the COM of the R121 backbone in 1 monomer and the corresponding position in the trimer, to compute the free-energy profile (Figs. 4 and S5).

The resulting free-energy landscape reveals a remarkably shallow energetic profile: the closed state (∼6.4 Å) and fully open state (∼11.0 Å) differ by only ∼1 kcal mol^−1^, with a similarly low intervening barrier, indicating comparable thermodynamic stability of the 2 states (Fig. S5d to f). This near-isoenergetic relationship between these end states is interrupted by a broad, shallow free-energy minimum at ∼7.7 Å, which defines a semi-open intermediate conformation. At this stage, the overall protein backbone predominantly adopts an open conformation, evidenced by a lower backbone root-mean-square deviation relative to the open state (0.703 Å, Fig. 4b, arrows) than to the closed state (1.557 Å, Fig. 4a, arrows) when the E120-R121 pair is excluded. Meanwhile, the entrance loop and the R121 side chain remain partially closed, resembling the semi-open conformation resolved in the crystal structure (PDB ID: 7DHQ) (Tan et al. 2021). The free energy landscape shows differences in the relative stability of the 3 conformational states; however, these relative energy levels may be sensitive to the simplified simulation setup, in which the pseudo-hexamer is modeled in isolation without neighboring shell components. Interactions within the native shell lattice may shift the relative stability of these states. Therefore, we emphasize that the energy barriers between the closed, semi-open, and open states are relatively low, rather than absolute ranking of state energies. The data suggest that the pore can readily interconvert between conformations, supporting a dynamic gating mechanism.

This intermediate conformation underscores the role of the E120-R121 pair as a molecular latch: their salt-bridge-like interactions stabilize the closed state and are disrupted during opening. The energy barriers separating this intermediate from either the closed or fully open state are low (∼ 1 to 2 kcal mol^−1^), reflecting primarily local side-chain rearrangements. Such small barriers indicate that modest thermal fluctuations under physiological conditions are compatible with frequent interconversions between the closed and open states on millisecond to sub-millisecond timescales. A similar gating mechanism has been reported for the central pore of the trimeric shell protein from a β-carboxysome lineage, CcmP, in which the open or closed state of the pore is controlled by the conformational rearrangement of 2 conserved residues, Glu69 and Arg70 (Larsson et al. 2017), which correspond to Glu145 and Arg146 in CsoS1D from *P. marinus* MED4, respectively. These findings suggest that electrostatic latch-mediated gating represents a conserved architectural principle across carboxysome shell proteins.

Instead of operating as a unidirectional transition triggered by ligand binding, channel gating of CsoS1D may function as a dynamic equilibrium, in which the trimers continuously fluctuate between closed and open states in response to thermal motion. Distinct from an induced-fit model in which ligand binding actively drives the pore opening (Changeux and Edelstein 2011), CsoS1D follows a conformational selection mechanism for metabolite transport: anionic metabolites (3-PGA and RuBP) bind preferentially during transiently sampled open conformations, and translocation is completed as the channel undergoes concerted motion toward the opposite open face, facilitated by the favorable electrostatic gradient within the pore. In this regime, metabolite flux is not rate-limited by the energetic cost of channel opening per se, but rather by the intrinsic metabolite capture kinetics and timescale of conformational fluctuations.

### Conserved residues underlying metabolite transport

To systematically identify the residues essential for metabolite translocation, we performed BLASTP-based sequence alignment of CsoS1D (PDB ID: 3FCH) against the UniProtKB/SwissProt database. Representative homologs were selected from both α- and β-carboxysomes, including CsoS1D and CcmP orthologs from phylogenetically diverse organisms: *P. marinus* MED4, *Halothiobacillus neapolitanus*, *Hydrogenovibrio crunogenus*, *Synechocystis* sp. PCC 6803, and *Synechococcus elongatus* PCC 7942, as well as structurally related BMC-T proteins (Figs. 4d and S6). Analysis of 33 representative homologs revealed that the R57, E120, R121, R178, and R229 residues are highly conserved across α-carboxysomes, β-carboxysomes, and other BMCs, strongly indicating their functional importance. Integrated analysis of conservation data and structural mapping reveals that these 5 residues form a multi-stage electrostatic pathway for metabolite passage traversing the entire pore axis from the cytosolic entrance to the luminal exit (Fig. 3): sequential recruitment (R57), gated entry (E120–R121), barrier modulation (R229), and internal anchoring (R178). The high sequence conservation of this transport pathway across taxonomically and functionally distinct carboxysome types and BMCs suggests that electrostatic-gated permeability represents a conserved and general transport principle for BMC-T channels. Further experimental validation through site-directed mutagenesis and functional assays is required.

## Discussion

The central pores of BMC shell proteins function as selective molecular filters that couple metabolite specificity with transport kinetics to regulate enzymatic flux and activity. BMC-T proteins, exemplified by CsoS1D in the α-carboxysome, possess enlarged pores that enable the translocation of bulky anionic metabolites essential for carboxysome functionality. In this study, we present a mechanistic analysis of CsoS1D permeability, examining in detail how this trimeric shell protein mediates the transport of 2 physiologically important but kinetically distinct metabolites, RuBP and 3-PGA, by integrating permeation dynamics, conformational dynamics, and sequence conservation analyses.

Our results suggest that CsoS1D can serve as a potential selective molecular gate that facilitates RuBP influx and 3-PGA efflux across the α-carboxysome shell. In contrast, the pores of hexameric CsoS1A and pentameric CsoS4A appear too narrow to accommodate these 2 bulky metabolites and are energetically unfavorable for their translocation, which is consistent with MD simulations of CcmK2 (Faulkner et al. 2020). Interestingly, recent computational simulations on a synthetic β-carboxysome shell showed the possibility of 3-PGA transiting through the BMC-H and BMC-P pores (Sarkar et al. 2024). Apart from the discrepancy in simulation systems (such as differences in protein composition, molecular crowding effects, water model parameterizations, and simulation timescales), the distinct observations suggest that metabolite transit across the carboxysome shell may involve multiple pathways with varying efficiencies; 3-PGA transport through BMC-H and BMC-P pores is not governed solely by pore confinement but likely reflects a subtle interplay between steric constraints, electrostatic interactions, and dynamic dehydration mechanisms. Consistent with our observations, RuBP has been found to exhibit much lower permeability through the BMC-H pore than other metabolites (Sarkar et al. 2024).

**Figure 5:**
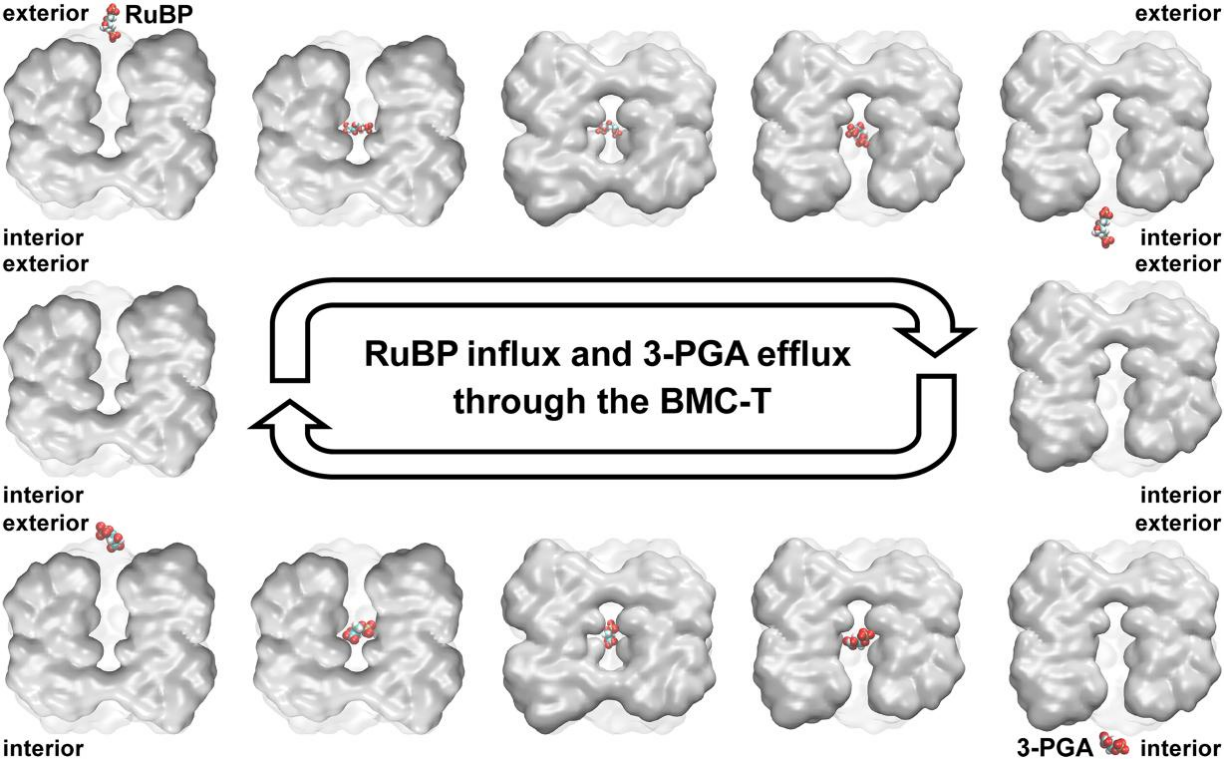
Proposed mechanistic model of the CsoS1D selective gating cycle. A schematic representation of the CsoS1D-mediated selective transport cycle within the BMC-T pore. RuBP influx is depicted at the top, while 3-PGA efflux is shown at the bottom. Driven by thermal fluctuations, the channel operates via a conformational selection mechanism, maintaining a dynamic equilibrium among closed, semi-open, and open states. The low energetic cost of these transitions (gating transition energy barrier of ∼2 kcal mol^−1^), as determined by TMD, allows CsoS1D to function as a flexible “molecular air-lock”, enabling rapid substrate-product exchange while preserving the internal microenvironment of the carboxysome.

Furthermore, we investigated the free-energy landscapes of RuBP and 3-PGA translocation through CsoS1D in unprecedented detail by employing TMD simulations and umbrella sampling. Our findings indicate a synchronized 3-stage gating mechanism for the translocation of 3-PGA and RuBP through CsoS1D driven primarily by electrostatic interactions and define an “air-lock” model for the CsoS1D gating cycle that reconciles high metabolic throughput with preservation of the carboxysome microenvironment (Fig. 5). In this mechanism, metabolites traverse a sequential pathway from the cytosol through the inter-trimer chamber and into the carboxysome lumen, and vice versa. This bidirectional flux is achieved through conformationally gated transitions of the CsoS1D pore between the open and occluded states under the conformational control of conserved pore-loop residues without compromising shell impermeability to small gas molecules. The flexible loops surrounding the central aperture operate asynchronously to create a transient vestibule, ensuring that a continuous channel fully open to the cytoplasm is difficult to construct, thereby preventing the back-diffusion of CO_2_. Our simulations expand the static crystal structure reported (Klein et al. 2009) to a dynamic transport mechanism that illustrates the full gating cycle of CsoS1D (Fig. 5). This “air-lock” strategy aligns with the proposed gating mechanism of the β-carboxysome analog CcmP (Cai et al. 2013; Larsson et al. 2017), suggesting a general gating mechanism across large-pore BMC-T shell proteins.

Interestingly, native α-carboxysomes from *H. neapolitanus* contain ∼3 CsoS1D trimeric pseudo-hexamers versus ∼975 CsoS1A/B/C hexamers, whereas native β-carboxysomes from *Synechococcus* 7942 contain 5 CcmP trimers (Sun et al. 2022, 2025). Despite this low abundance, the relatively low free-energy barrier of CsoS1D (on the order of ∼2 kcal/mol) suggests substantially higher per-pore transport efficiency (Hänggi et al. 1990; Berezhkovskii and Bezrukov 2005). Consistent with this, our sRAMD simulations show that CsoS1D exhibits remarkably higher passage probabilities than CsoS1A and CsoS4A, particularly for RuBP (Table 1). Thus, the enlarged pore diameter of CsoS1D (10.9 to 12.4 versus 3.8 to 5.8 Å for BMC-H/P, Fig. 1) and its higher translocation probability (Table 1) support its role as a potential conduit for bulky metabolites and an important contributor to overall metabolite flux across the shell.

However, the barrier differences between CsoS1D and CsoS1A/CsoS4A are only on the order of a few kcal/mol. Because transport rates depend exponentially on the free-energy barrier, such differences may compensate for orders-of-magnitude differences in copy number, but they may not fully offset the large stoichiometric imbalance. Therefore, metabolite transport across the shell is likely mediated by multiple pathways with distinct efficiencies, and the contribution of the more abundant shell proteins should not be overlooked. Indeed, deletion of CsoS1D did not result in a strong high-CO_2_-requiring phenotype (Desmarais et al. 2019), suggesting that the loss of this highly efficient conduit may be partially compensated by other, more abundant but less permeable BMC-T and BMC-P shell proteins.

The findings of CsoS1D and CcmP established tandem BMC-domain trimers as specialized, efficient but low-copy shell components with stacked, asymmetrically gated pores. Intriguingly, the free-energy landscape governing channel gating is remarkably shallow (Fig. 2), indicating that thermal energy alone is sufficient to drive frequent and reversible transitions among the open, closed, and semi-open pore conformations. Rather than requiring ligand-induced activation, this energetic architecture permits a conformational selection mechanism wherein CsoS1D constitutively samples a conformational ensemble, with metabolite translocation occurring opportunistically during transiently accessible open states.

Our results further elucidate the specific roles of the key conserved residues at the CsoS1D pore in orchestrating the 3-stage electrostatic transport mechanism. The R57 residue positioned at the outer pore entrance mediates the initial recruitment of negatively charged 3-PGA and RuBP through electrostatic interactions with their anionic phosphate groups. R121 and E120 form a conserved gating node that regulates metabolite flux, whereas E120 simultaneously acts as an electrostatic repulsive barrier preventing uncontrolled metabolite flux. This arrangement permits controlled, gated transport rather than passive diffusion. R229 couples electrostatically with E120 to fine-tune the effective free-energy barrier height, acting as an allosteric regulator of gating stringency in response to metabolite concentration or cellular signaling. R178 located at the trimer-of-dimer interface stabilizes ligands in the internal electropositive binding pocket (identified in Stage III of the permeation pathway), facilitating transient stabilization and controlled release toward the luminal exit.

Our findings highlight the central role of electrostatic potentials in governing metabolite permeation through carboxysome shell pores. Because electrostatic interactions are inherently sensitive to protonation states, channel permeability and selectivity are expected to depend on pH-dependent charge distributions of both pore-lining residues and transported metabolites. In this study, all simulations were performed at a cytoplasmic pH of 7.96, which corresponds to the physiological conditions under which carboxysomes operate. Future work incorporating constant-pH or continuous-pH MD simulations will be essential to elucidate how shifts in protonation equilibria modulate free-energy landscapes and transport kinetics under varying intracellular conditions (Barroso daSilva and Dias 2017; Radak et al. 2017). Such effects, although beyond the scope of the current study, represent an important dimension for understanding and engineering selective transport in BMCs.

More broadly, CsoS1D belongs to the BMC-T family, which is highly conserved across α-carboxysomes, β-carboxysomes, and diverse metabolosomes (Melnicki et al. 2021). The β-carboxysome BMC-T homolog CcmP exhibits similar electrostatic pore architecture (Kerfeld and Melnicki 2016; Larsson et al. 2017). Both CsoS1D and CcmP, despite displaying low abundance in the carboxysome shell, likely evolved for high-efficiency transport of bulky metabolites that sterically exclude from abundant BMC-H and BMC-P proteins. Notably, many metabolosome lineages, including sugar-phosphate-utilizing (SPU) BMCs that transport phosphorylated intermediates such as glyceraldehyde-3-phosphate, DHAP, and lactaldehyde, encode CsoS1D-like BMC-T proteins despite lacking CO_2_-concentrating functions. The strong conservation of the electrostatic pathway residues identified (R57, E120, R121, R178, and R229) across diverse BMC-T proteins (Figs. 4d and S6) suggests that charge-based recruitment, gating, and release of anionic metabolites and dynamic pore control of BMC-T proteins may represent the generalizable design principles for BMC shell permeability. In this context, the CsoS1D “air-lock” mechanism provides a mechanistic framework for understanding how BMC-T proteins across diverse metabolic modules regulate flux of bulky, highly charged metabolites while maintaining shell integrity.

It is worth noting that in the present simulations, the shell proteins were modeled in isolation with solvent-exposed boundaries under periodic boundary conditions, following approaches adopted in previous studies (Mahinthichaichan et al. 2018; Faulkner et al. 2020), rather than embedded within an explicit 2D shell lattice or 3D shell protein assemblies. Backbone restraints were applied to the pore region to preserve the experimentally resolved local fold and to minimize artifacts associated with solvent exposure at the truncated boundaries. While this setup provides a reasonable approximation of the intrinsic transport properties of the pore, it does not fully capture the mechanical and energetic constraints imposed by neighboring shell proteins in the native environment. For instance, lateral packing may affect large-scale conformational changes associated with pore opening and closing, which involve peripheral structural elements. As such, the observed dynamics reflects the intrinsic flexibility and gating propensity of an isolated CsoS1D protein, rather than a quantitatively complete description of its physiological behavior within the intact shell. Nevertheless, relative differences in transport propensity and barrier profiles between pore types are expected to remain qualitatively robust, as these are primarily governed by local pore geometry and interactions. Future simulations incorporating explicit shell lattices, informed by ongoing advances in high-resolution structural characterization of native carboxysomes, will be required for capturing these collective effects and further refining the quantitative description of metabolite transport.

In summary, this study reveals the mechanistic basis for selective metabolite translocation through the CsoS1D pore across the α-carboxysome shell. CsoS1D operates via a 3-stage “air-lock” electrostatic transport mechanism, permitting the passage of RuBP and 3-PGA during transient permissive conformations. These findings provide a framework for understanding the natural permeability of BMC shells and rationally tuning BMC permeability for optimizing their metabolic flux and functions in synthetic biology to underpin crop engineering and diverse biotechnological applications.

## Materials and methods

### Model construction and molecular dynamics simulation

Crystal structures of α-carboxysome shell proteins were obtained from the Protein Data Bank (PDB): the trimeric shell (BMC-T) protein CsoS1D (PDB ID: 3FCH, 2.20 Å) (Klein et al. 2009), the pentameric shell (BMC-P) protein CsoS4A (PDB ID: 2RCF, 2.15 Å) (Tanaka et al. 2008), and the hexameric shell (BMC-H) protein CsoS1A (PDB ID: 2EWH, 1.40 Å) (Tsai et al. 2007). As no BMC-T structure with 2 open pores was available, the asymmetric “one-open/one-closed” CsoS1D structure (3FCH) was used (Klein et al. 2009). Protonation states at pH 7.96 were assigned using PropKa (Olsson et al. 2011; Huang et al. 2022), with CYS166B and LYS222B modeled as CYN and LYN, respectively. Proteins were described using the Amber ff14SB force field (Maier et al. 2015).

The ligands 3-PGA and RuBP were built in GaussView (Dennington et al. 2016) and optimized at the DFT B3LYP/6-31G(d) level with Gaussian 16 (Frisch et al. 2016). The atomic charges of ligands were calculated using the restricted electrostatic potential method at the HF/6-31G(d) level using Gaussian 16. Force field parameters for the ligands were generated based on the Generalized Amber Force Field using the Antechamber module in AmberTools (Case et al. 2023).

Six protein-ligand systems were constructed by placing each ligand 15 Å above the pore entrance of its corresponding shell protein (Dror et al. 2012). Systems were solvated in truncated hexahedral TIP3P water boxes with a 10 Å buffer (Maier et al. 2015), neutralized, and supplemented with K^+^/Cl^−^ ions to 0.15 M. The detailed composition of each simulation system is summarized in Table S1.

For the TMD simulation systems (initial and target structures), a periodic water box was built with a 12 Å extension of TIP3P water molecules around the protein surface. To balance the system charge and achieve an ionic strength of 0.15 M, 74 Na^+^ and 65 Cl^−^ ions were added. The resulting systems contained a total of 58,237 atoms, with simulation box dimensions of 105.118 Å × 100.469 Å × 68.277 Å.

Energy minimization was performed using 1,000 steps of steepest descent followed by 1,000 steps of conjugate gradient minimization. Systems were heated from 0 to 310 K over 100 ps and equilibrated for 20 ns under NVT and 20 ns under NPT conditions, with backbone restraints of 10 kcal mol^−1^ Å^−2^. Ligand positions were restrained along the pore axis using a harmonic potential during equilibration. All simulations were carried out with NAMD 2.14 using periodic boundary conditions (Phillips et al. 2005). Temperature (310 K) and pressure (1 atm) were maintained using Langevin dynamics and the Nosé-Hoover Langevin piston, respectively. Long-range electrostatics were treated with PME, covalent bonds to hydrogen were constrained with SHAKE, and a 2-fs time step was used. For each protein-ligand system, 50 ns of equilibrium simulation was performed, yielding a total of 300 ns across all permeation systems. Additional 50 ns equilibrations were conducted for the open and closed CsoS1D systems used in TMD.

### s-RaMD-MD simulations and Umbrella sampling for characterizing transport free-energy landscapes

To enhance ligand sampling efficiency beyond conventional RaMD-based approaches, self-referenced random acceleration MD (s-RaMD-MD) simulations were performed, in which the ligand itself served as the reference frame (Mao et al. 2024). From the final 10 ns of NPT equilibration, 50 snapshots were extracted at 0.2 ns intervals and used as initial structures. For each ligand-protein combination, 50 independent replicas were generated, yielding a total of 300 s-RaMD-MD simulations. For RuBP, the phosphate group closest to the channel entrance was selected as the force-application site, while the ligand COM was used as the reference point. For 3-PGA, the single phosphate group was defined analogously. In the s-RaMD-MD simulations, the external force was applied to the phosphate group proximal to the pore rather than to the COM of the substrate. This choice was motivated by the fact that the molecular dimensions of 3-PGA and RuBP exceed the pore diameter, such that applying force to the COM could lead to steric trapping and hinder successful translocation. In addition, both substrates contain phosphate groups that are expected to play a key role in interactions with the pore environment, making them a physically relevant handle for guiding entry. We note that this setup may introduce a degree of orientation bias in how the substrates approach the pore. Therefore, the results should be interpreted as reflecting force-assisted entry pathways under these constraints, rather than fully unbiased diffusion behavior. Using the phosphate group as the pulling site also ensures consistency across both substrates, facilitating direct comparison of their translocation behavior. The s-RaMD-MD distance threshold was set to 0.01 Å, with acceleration strengths of 0.2 and 0.3 kcal mol^−1^ Å^−1^ amu^−1^ applied. Ligand motion was confined within a cylindrical region (radius 20 Å) along the channel axis using a harmonic wall potential (10 kcal mol^−1^ Å^−2^). Each replica consisted of alternating 200 fs cycles of s-RaMD and conventional MD, for a total of 1 ns per replica, corresponding to an aggregate sampling time of 600 ns.

Umbrella sampling simulations were subsequently performed to quantify the free-energy landscapes associated with ligand permeation through BMC-T, BMC-P, and BMC-H shell proteins. Initial configurations for each umbrella window were extracted from the s-RaMD-MD trajectories. The reaction coordinate was defined as the ligand displacement along the channel axis (*z*-direction), with the initial window positioned at a distance of 15 Å from the protein surface. Umbrella windows were initially spaced at 0.4 Å intervals, with the protein backbone restrained using a harmonic potential of 10 kcal mol^−1^ Å^−2^. A harmonic bias of 5 kcal mol^−1^ Å^−2^ was applied in each window, and simulations were run for 10 ns per window. In regions with insufficient overlap between adjacent windows, additional windows were introduced at 0.2 Å spacing, and the biasing force constant was increased to 30 kcal mol^−1^ Å^−2^. Each additional window was also simulated for 10 ns. In total, each system comprised 145 umbrella windows, corresponding to 2.9 μs of cumulative sampling. Free-energy profiles were reconstructed using the weighted histogram analysis method (WHAM) (Kumar et al. 1992).

As no fully open BMC-T structure is currently available, simulations were based on the CsoS1D structure containing 1 open and 1 closed pore (Klein et al. 2009). Exploiting the structural symmetry of the CsoS1D dimer, ligand permeation was assumed to proceed in 2 symmetric steps: from the exterior into the central interface through 1 subunit, and subsequently from the interface to the opposite side. Accordingly, the unilateral permeation free-energy profile obtained from simulations was symmetrically extended to represent the complete translocation process across the trimeric dimer.

### TMD simulations and Umbrella sampling of the free-energy barrier for the closed-to-open transition

After equilibration, TMD simulations were performed to drive the closed-to-open conformational transition of the CsoS1D monomer (Schlitter et al. 1994). Because the backbone conformation at the bottom of the lateral channel entrance is identical in the closed and open states, backbone atoms within 6 Å of this region were restrained with a harmonic potential (10 kcal mol^−1^ Å^−2^) to prevent overall protein displacement without affecting the gating motion. A 1-μs TMD simulation was then carried out to transform the closed-state monomer toward the open-state target structure. All other simulation parameters were identical to those used in conventional MD simulations.

Umbrella sampling was subsequently employed to quantify the free-energy profile of the closed-open transition. Initial structures for each window were extracted from the TMD trajectory. The reaction coordinate was defined as the distance between the centroid of the backbone C and N atoms of the gating residue R121 in a single subunit and the corresponding centroid in the trimeric reference structure. The coordinate ranging from 5.86 to 13.07 Å was divided into 50 windows. Window selection was guided by both the reaction coordinate value and backbone displacement along the gating pathway to ensure continuous conformational sampling. During umbrella sampling, the same backbone restraint was maintained, and a harmonic biasing potential with a force constant of 100 kcal mol^−1^ Å^−2^ was applied along the reaction coordinate. Each window was simulated for 10 ns. Simulations were performed for all 3 subunits, yielding a total sampling time of 1.5 μs. Free-energy profiles were reconstructed using the WHAM, and profiles from the 3 subunits were averaged to obtain the final free-energy landscape of the conformational transition.

### Sequence retrieval, filtering, and conservation analysis

The retrieval of multiple homologous sequences was conducted by BLASTP and UniProt (Altschul et al. 1997). Five individual BLASTP searches were performed within UniProt Reference Clusters 90 (UniRef90) database by using CsoS1D from *P. marinus* MED4, CsoS1D from *H. neapolitanus*, CcmP from *S. elongatus* PCC7942, BMC-T2 and BMC-T3 from *Haliangium ochraceum* DSM14365 as reference queries, respectively. The UniRef90 database, which clusters sequences above 90% identity to reference query as a threshold, was chosen to remove redundant protein sequences in case of any taxonomy bias against some specific organisms and ensure alignment quality. Each query was also performed with 250 hits to emphasize biodiversity. Sequences of CsoS1D-like shell proteins from sugar phosphate-processing BMCs (SPU-BMCs) were included. These non-redundant homologous sequences were then aligned using Cluster Omega (https://www.ebi.ac.uk/) and output as a FASTA file for sequence logo generation. Multiple sequence alignments were visualized with WebLogo 3.9.0 (https://weblogo.threeplusone.com/) to generate the final sequence logo, highlighting the strictly conserved potential functional residues. To highlight biodiversity and reduce non-redundancy, representative CsoS1D-like, CcmP-like and BMC-T-like sequences were selected across Family/Genus/Species levels with each taxa retains around 10 sequences. Multiple sequence alignments were visualized with ESPript 3.2 (Robert and Gouet 2014), highlighting fully and partially conserved residues relative to CsoS1D.

## Supplementary Material

kiag331_Supplementary_Data

## Data Availability

The protein structures used in this study are accessible from the Protein Data Bank (PDB codes: 3FCH, 2RCF, and 2EWH). The data supporting the findings of this study are available from the corresponding author upon request. The data are not publicly available due to privacy or ethical restrictions.
