# Supplementary material for: “Air-Lock” gating mechanism of CsoS1D for metabolite translocation through the α-carboxysome shell
Source: Plant Physiol. 2026 Jun 3;201(2):kiag331. doi: 10.1093/plphys/kiag331 (PMC13316943; doi:10.1093/plphys/kiag331)
Supplement: kiag331_Supplementary_Data [file kiag331_supplementary_data.zip › SI_CsoS1D_R1_clean.docx]

Supplementary Information

for

**“Air-Lock” gating mechanism of CsoS1D for metabolite translocation through the α-carboxysome shell**

Quan Wen^1,#^, Yue Wang^1,#^, Guo-Can Huang^1^, Hong-Yu Pan^1^, Yue-Yang Tang^2^, Li-Hua Bie^1^, Lu-Ning Liu^1,2*^, Jun Gao^1,*^

^1^ Hubei Key Laboratory of Agricultural Bioinformatics, College of Informatics, Huazhong Agricultural University, Wuhan 430070, China

^2^ Institute of Systems, Molecular and Integrative Biology, University of Liverpool, Liverpool L69 7ZB, UK

^#^These authors contribute equally.

*Corresponding authors: gaojun@mail.hzau.edu.cn; luning.liu@liverpool.ac.uk


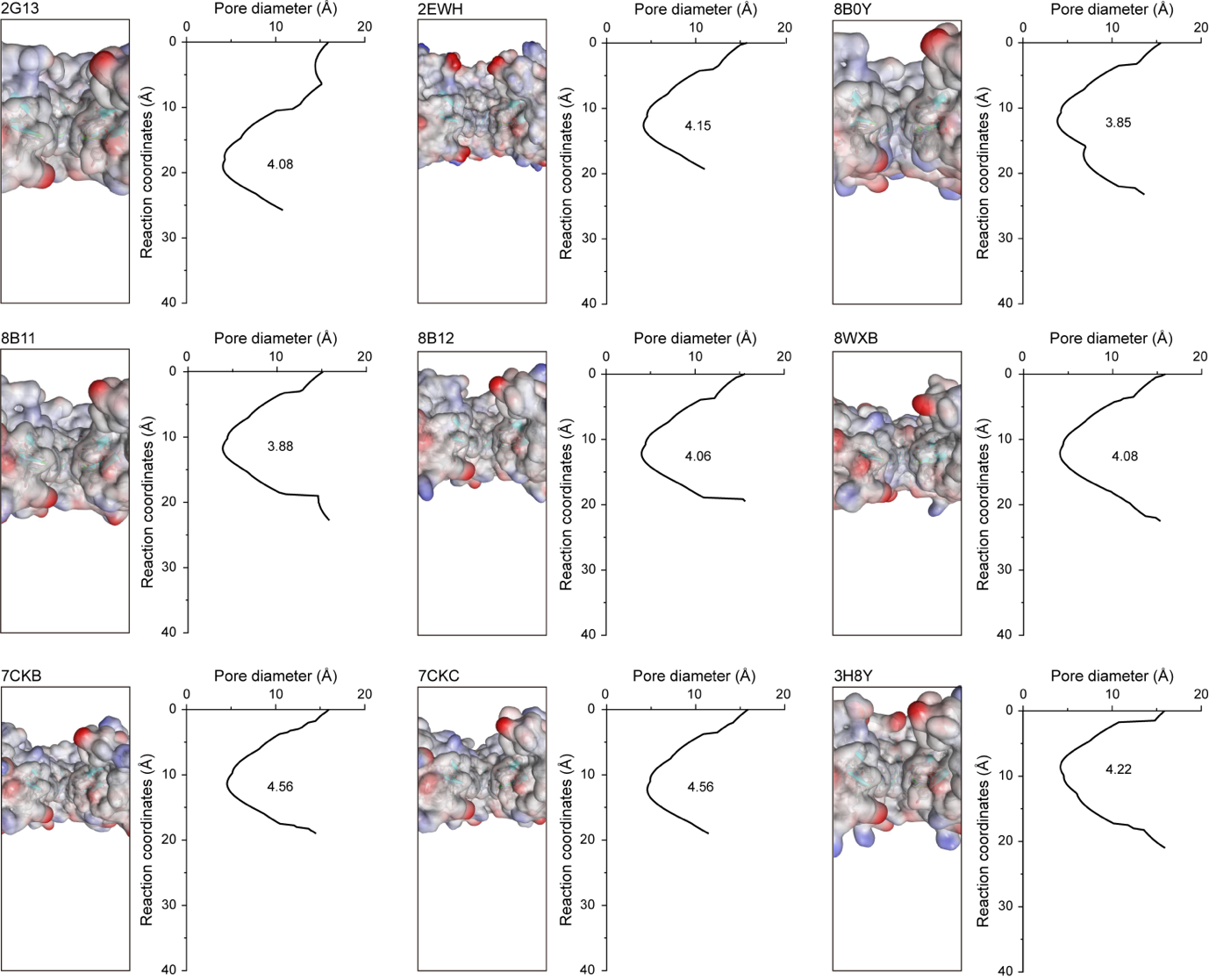


**Figure S1. Structural representations and pore diameter profiles of nine hexameric shell proteins (BMC-H).** Each panel displays the crystal structure of a BMC-H hexamer alongside its calculated pore dimensions to illustrate channel accessibility. The structures were retrieved from the Protein Data Bank (PDB), with the following PDB IDs: 2G13, 2EWH, 8B0Y, 8B11, 8B12, 8WXB, 7CKB, 7CKC, and 3H8Y. These profiles demonstrate the varying degrees of pore constriction observed across different BMC-H homologs.


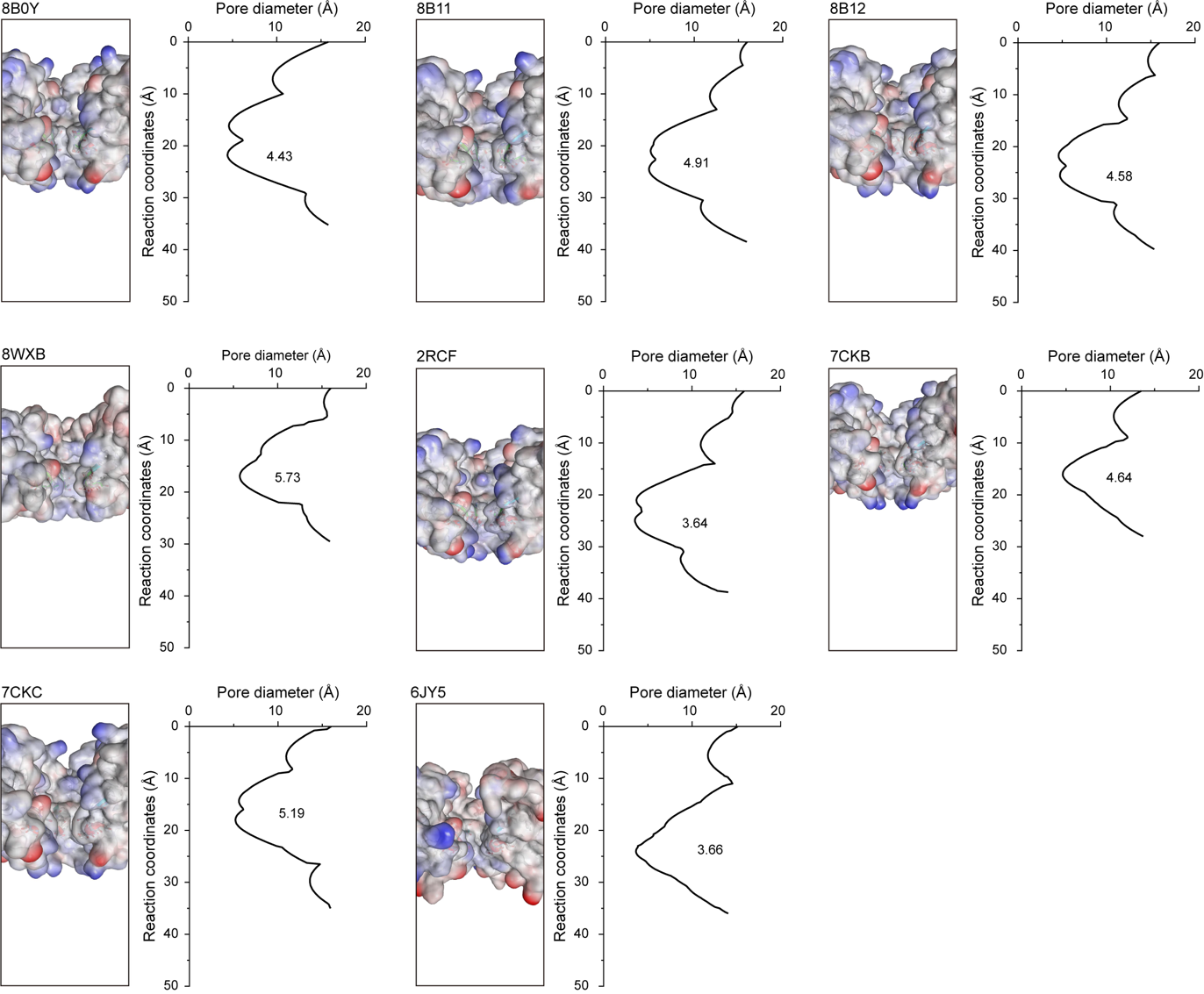


**Figure S2. Structural representations and pore diameter profiles of eight pentameric shell proteins (BMC-P).** Each panel displays the crystal structure of a BMC-P pentamer alongside its calculated pore dimensions. The structures were retrieved from the Protein Data Bank (PDB), with the following PDB IDs: 8B0Y, 8B11, 8B12, 8WXB, 2RCF, 7CKB, 7CKC, and 6JY5. These profiles illustrate the channel characteristics of the pentameric protein that typically form the vertices of the BMC shell.


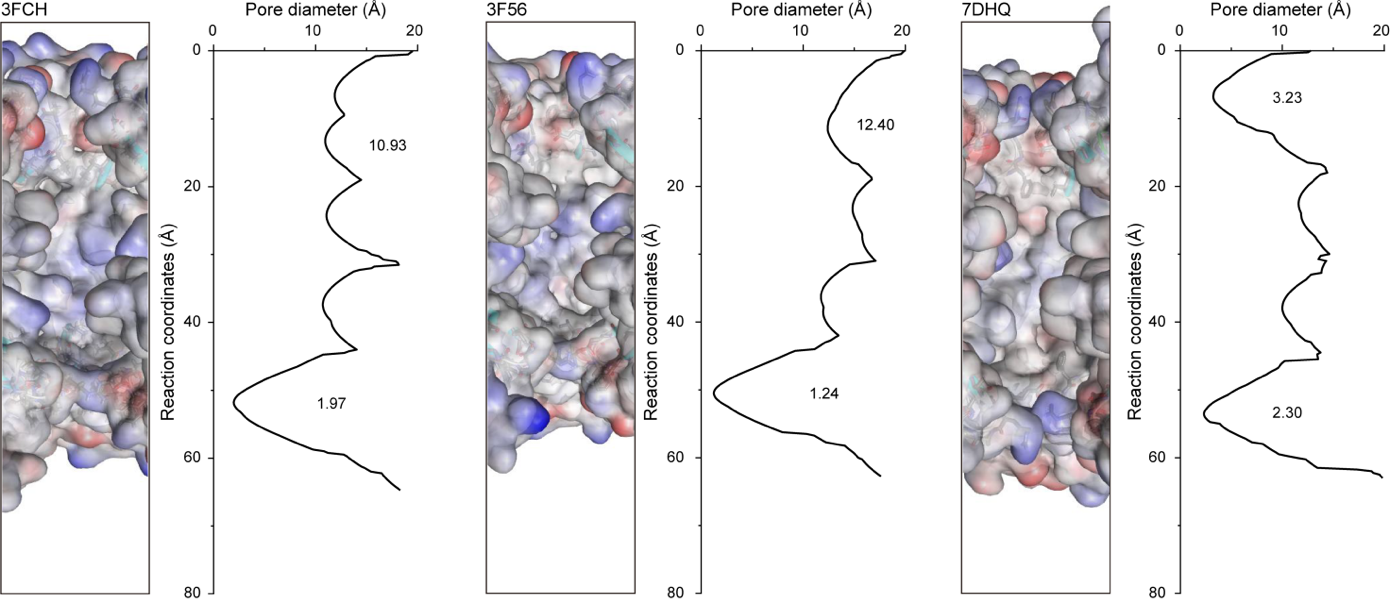


**Figure S3. Structural representations and pore diameter profiles of three trimeric shell proteins (BMC-T).** Each panel displays the crystal structure of a BMC-T trimer alongside its calculated pore dimensions. The structures were retrieved from the PDB database, with the following PDB IDs: 3FCH, 3F56, and 7DHQ. Notably, PDB ID 3FCH corresponds to the CsoS1D structure used as the primary model in this study.


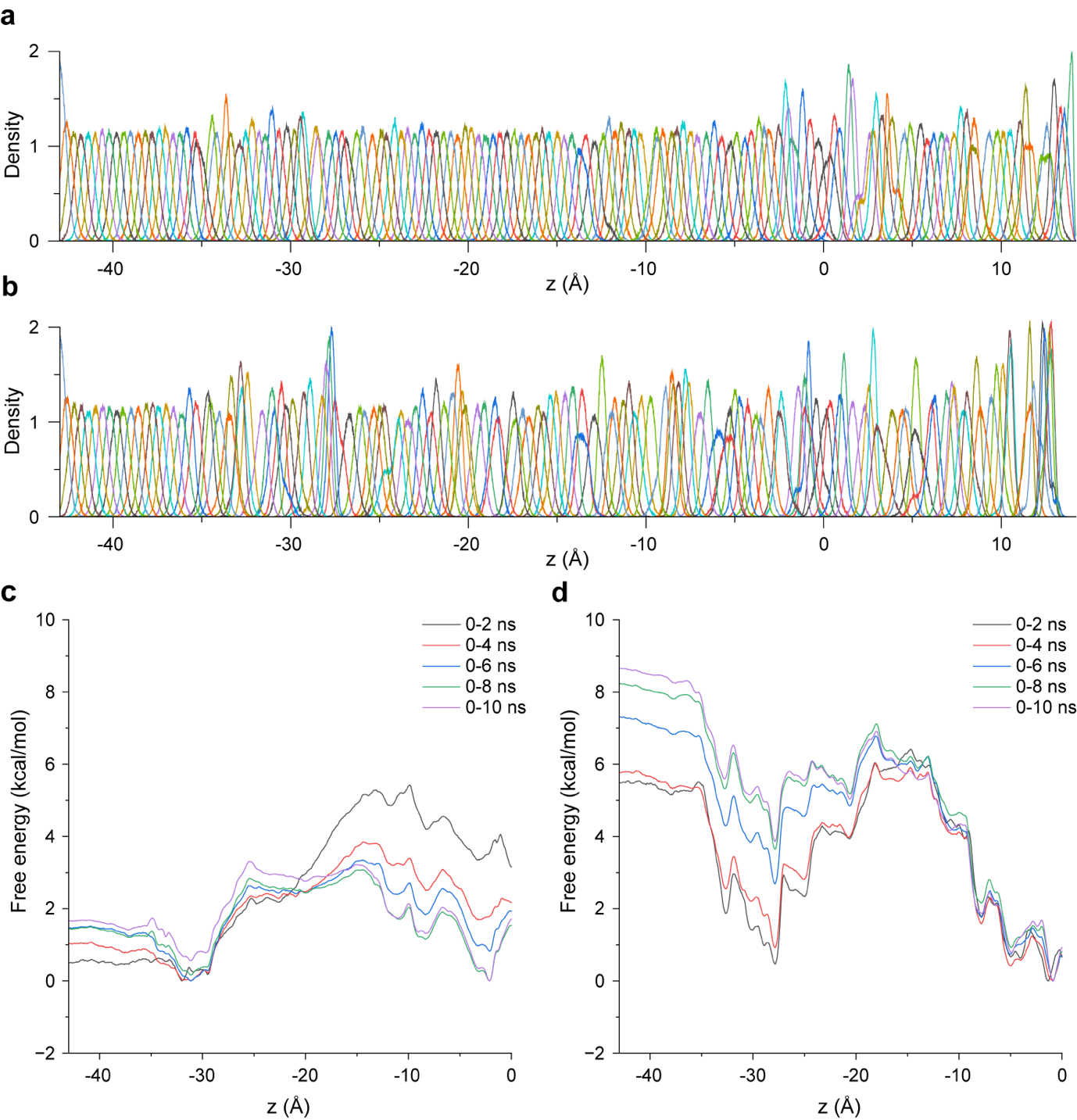


**Figure S4. Sampling quality assessment and convergence analysis of umbrella sampling simulations for 3-PGA and RuBP permeation through CsoS1D.** a, b) Histogram distributions of the reaction coordinate (z-axis) for 3-PGA (a) and RuBP (b). The sufficient overlap between adjacent umbrella windows ensures continuous sampling along the reaction pathway. c, d) Time-dependent evolution of the free energy profiles for 3-PGA (c) and RuBP (d). Profiles were calculated over cumulative simulation time intervals (0-2 ns, 0-4 ns, 0-6 ns, 0-8 ns, and 0-10 ns). The stabilization of the profiles with increasing simulation time indicates that the free energy calculations have reached convergence.


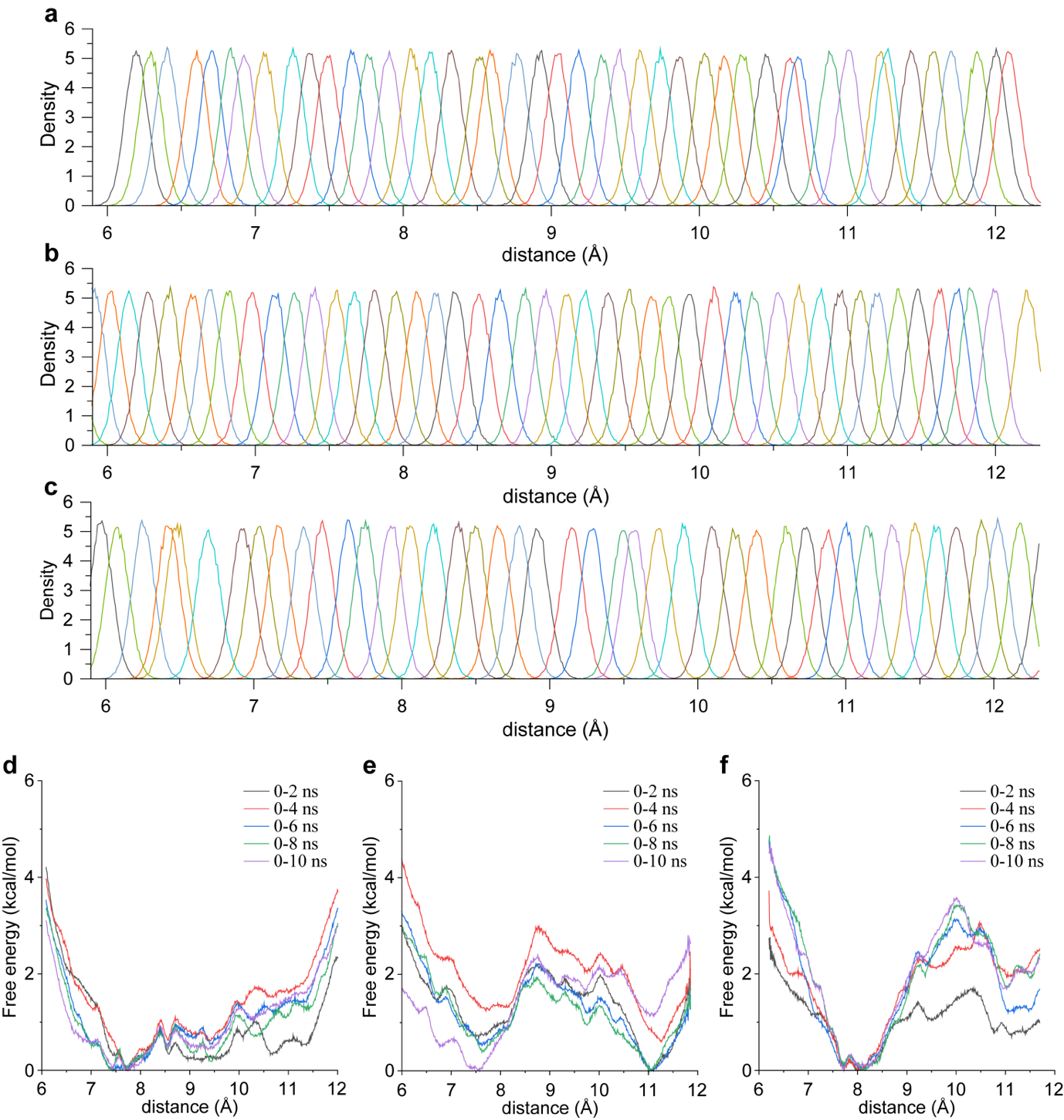


**Figure S5. Sampling quality assessment and convergence analysis of umbrella sampling simulations for conformational transitions of the three individual subunits.** (a-c) Histogram distributions of the reaction coordinate, defined by the R146 backbone displacement, for the three subunits A-C of CsoS1D. Sufficient overlap between adjacent umbrella windows ensures continuous sampling along the transition pathway. (d-f) Time-dependent evolution of the free energy profiles for Subunit A-C. Free energy profiles were calculated over cumulatively increasing simulation time intervals (0-2 ns to 0-10 ns), demonstrating that the free-energy calculations for all three subunits have reached convergence.


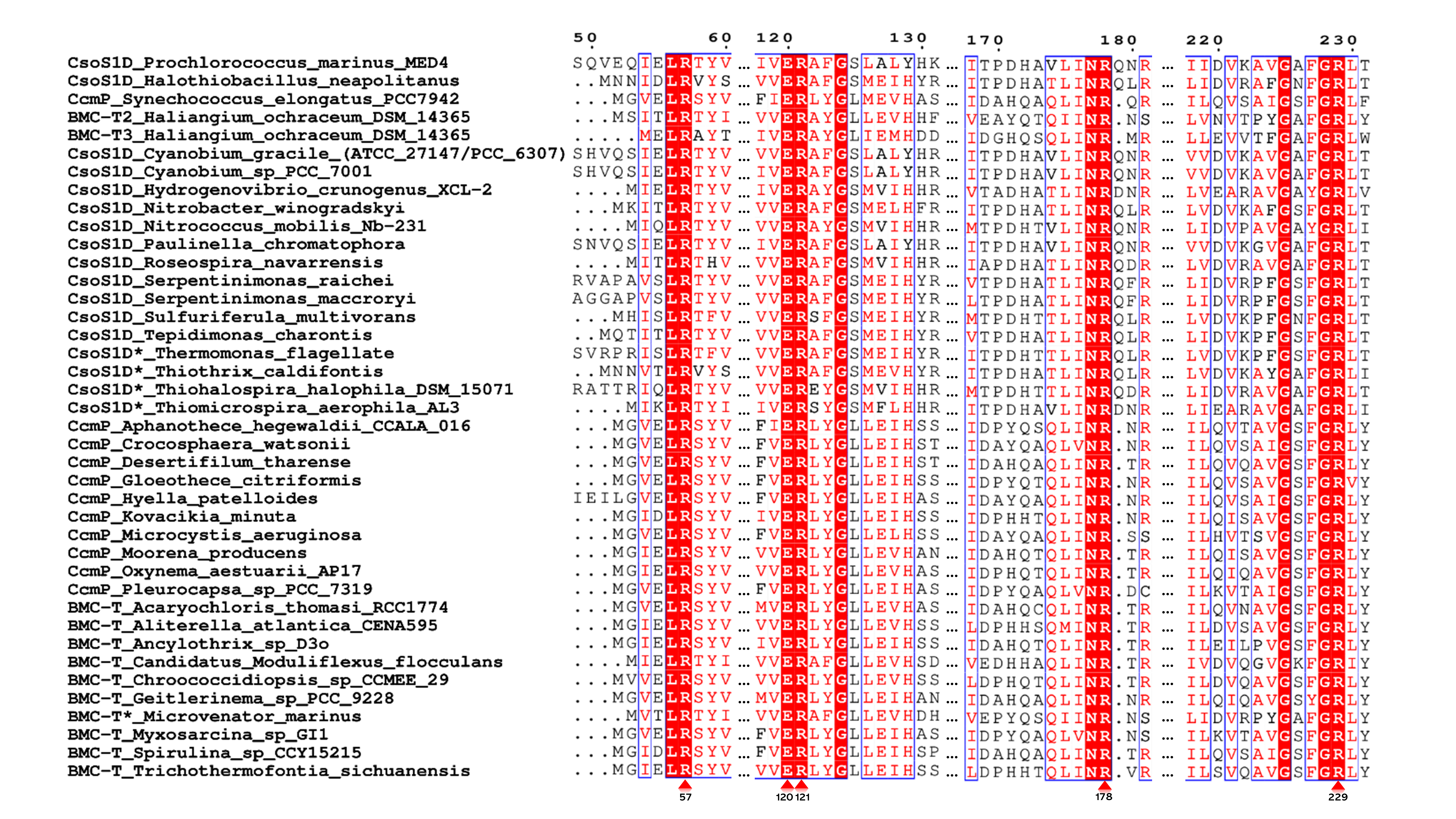


**Figure S6. Multiple sequence alignment of CsoS1D homologs among different species.** Residues that are fully conserved across all homologs are shown in white font on a red background, whereas residues with high similarity are shown in red font on a white background; blue boxes denote regions of high sequence similarity. Sequences marked with an asterisk (*) on CsoS1D represent CsoS1D-like shell proteins in sugar phosphate-processing bacterial microcompartments (SPU-BMCs). See also Figure 4d.

**Table S1. Detailed composition of the molecular dynamics simulation systems.** The table summarizes the setup of each ligand-protein complex, including systems containing 3-PGA or RuBP with CsoS1A, CsoS4A, and CsoS1D. The parameters include the total number of atoms, the dimensions of the periodic boundary box (X, Y, Z lengths in Å), and the number of potassium (K^+^) and chloride (Cl^-^) ions added to neutralize the system and mimic physiological ionic strength.

| **Systems** | **Total Atoms** | **Box Length (Å)** | | | **Number of Ions** | |  |
| --- | --- | --- | --- | --- | --- | --- | --- |
|  |  | X | Y | Z | K⁺ | Cl⁻ | |
| 3-PGA-CsoS1A | 157814 | 129.88 | 129.28 | 108.97 | 175 | 166 | |
| RuBP-CsoS1A | 162217 | 129.88 | 129.28 | 111.30 | 180 | 170 | |
| 3-PGA-CsoS4A | 135964 | 116.64 | 118.87 | 112.87 | 154 | 150 | |
| RuBP-CsoS4A | 141658 | 116.64 | 118.87 | 117.34 | 155 | 150 | |
| 3-PGA-CsoS1D | 203453 | 131.84 | 127.97 | 138.19 | 244 | 220 | |
| RuBP-CsoS1D | 211484 | 131.84 | 127.97 | 143.37 | 245 | 220 | |

**Movie S1.** Representative trajectory illustrating the permeation of 3-PGA through the CsoS1D channel.

**Movie S2.** Representative trajectory illustrating the permeation of RuBP through the CsoS1D channel.

**Movie S3.** Side-view representation of the conformational transition of a CsoS1D protomer from the closed to the open state.

**Movie S4.** Top-view representation of the conformational transition of a CsoS1D protomer from the closed to the open state.
